# Community Regulation: The Relative Importance of Recruitment and Predation Intensity of an Intertidal Community Dominant in a Seascape Context

**DOI:** 10.1371/journal.pone.0023958

**Published:** 2011-08-26

**Authors:** Gil Rilov, David R. Schiel

**Affiliations:** Marine Ecology Research Group, School of Biological Sciences, University of Canterbury, Christchurch, New Zealand; National Institute of Water & Atmospheric Research, New Zealand

## Abstract

Predicting the strength and context-dependency of species interactions across multiple scales is a core area in ecology. This is especially challenging in the marine environment, where populations of most predators and prey are generally open, because of their pelagic larval phase, and recruitment of both is highly variable. In this study we use a comparative-experimental approach on small and large spatial scales to test the relationship between predation intensity and prey recruitment and their relative importance in shaping populations of a dominant rocky intertidal space occupier, mussels, in the context of seascape (availability of nearby subtidal reef habitat). Predation intensity on transplanted mussels was tested inside and outside cages and recruitment was measured with standard larval settlement collectors. We found that on intertidal rocky benches with contiguous subtidal reefs in New Zealand, mussel larval recruitment is usually low but predation on recruits by subtidal consumers (fish, crabs) is intense during high tide. On nearby intertidal rocky benches with adjacent sandy subtidal habitats, larval recruitment is usually greater but subtidal predators are typically rare and predation is weaker. Multiple regression analysis showed that predation intensity accounts for most of the variability in the abundance of adult mussels compared to recruitment. This seascape-dependent, predation-recruitment relationship could scale up to explain regional community variability. We argue that community ecology models should include seascape context-dependency and its effects on recruitment and species interactions for better predictions of coastal community dynamics and structure.

## Introduction

A recent paper [1] identified “predicting the strength and context-dependence of species interactions across multiple scales” as one of three core areas in the “frontiers of ecology”. The present study specifically addresses this type of inquiry because it tests species interactions at two context-dependent attributes of the system: the local landscape and its effect on the predator guild, and the recruitment rates of a dominant prey species. In an effort to understand the structure and dynamics of marine communities, the relationships between prey recruitment and abundance, predator abundance, and species interaction strength have been intensely studied in the last few decades. The importance of recruitment rates of dominant prey species to their local abundance [supply–side ecology, sensu 2] has been demonstrated in many studies, and both positive and negative effects of recruitment rate on adult numbers were demonstrated [e.g.], [ 3], [4,5]. Predation on dominant organisms is also known to be highly important in shaping community structure in marine communities [e.g.], [ 6], [7], [8], [9], [10], [11,12]. One important challenge for community ecologists, however, is understanding the context that determines the relative importance of recruitment and predation on the population size of dominant species and therefore of community structure as a whole.

Some models of coastal community organization and dynamics and some empirical studies indicate that there is a strong positive relationship between rates of recruitment of basal, benthic, space-occupying species (e.g., barnacles and mussels), and per-capita consumption intensity on their recruits and adults in the context of the regional oceanography [e.g.], [ 3], [13], [14], [15,16]. This “benthic-pelagic” or “bottom-up/top-down” coupling [13], [17], [18], [19], [20] can occur because the recruitment of many benthic-dwelling, macro-predators (e.g., sea stars and some whelks) is presumably influenced by the same oceanographic forcing as their prey [indirect causation], [ 13,see 21 for a coral reef fish example] and probably also because the presence and activity of these predators depend greatly on the local food supply [numerical and functional response], [ i.e., direct causation, e.g., 14,22]. Data from the Pacific coast of North and South America show that the life history of intertidal predators (i.e., whether or not they have a pelagic larval stage) influences the relationship between prey recruitment and predator abundance, and, in contrast to the other examples, a positive correlation exists only in predators lacking pelagic larvae but not with those with such larvae [23].

A key to the understanding of the relationship between prey recruitment and abundance and predation intensity on it is to measure these parameters on both small (hundreds of meters) and large (hundreds of kilometers) spatial scales. The combination of large-scale and small-scale studies adds power to, and increases confidence in, inferences about the functional dynamics of ecological communities [24], [25]. We therefore tested the relationship between predation intensity and prey availability and recruitment using multiple sites in the North and South Islands of New Zealand. We focus our investigation on mussels as prey because (1) they are community dominants on temperate shores in many biogeographic regions, (2) they are important facilitators for other benthic species and (3) they are a major food source in the ecosystem [26].

Our study extends on earlier work that demonstrated the importance of seascape (intertidal reefs with or without nearby subtidal reefs) to predation intensity in the intertidal zone through the presence or absence of subtidal predators (fish and crabs) [27], [28]. Those studies showed that at sites where intertidal benches are contiguous with kelp-covered subtidal reefs (reef-to-reef seascapes, hereafter R-R sites) in southeastern New Zealand, the primary predators were highly mobile labrid fishes and crabs that moved from subtidal reefs during high tide and ate low- and to a lesser degree mid-intertidal juvenile mussels [28]. Predation on juvenile mussels was strong at such east-coast R-R sites, but weak on intertidal rocky benches with shallow, sandy, subtidal bathymetry (reef-to-sand seascapes, hereafter, R-S sites). Data collected in a small-scale (2 km shoreline) short-term (2-mo) recruitment-evaluation effort during that study indicated that local recruitment rates of mussels may be much higher at R-S seascapes. This suggested that in this system there may be a disconnection between prey availability (mussel recruitment and abundance) and predation intensity (the abundance and activity of large mobile consumers that prey on mussels). However, that data was too limited, both temporally and spatially, to enable meaningful testing of the relationship between prey recruitment and predation intensity in the seascape context.

In the present study, we target this relationship and tested the relative importance of predation and recruitment to intertidal adult mussel populations and the hypothesis that predation intensity and recruitment rates were indeed unrelated. This was done in the context of seascape (intertidal reefs with and without nearby subtidal reefs: R-R and R-S seascapes) and at two spatial scales: small (2–3 km: area) and large (the North and South Islands). Specifically, we measured the relationships among the following parameters: prey abundance (mussel percent cover), average mussel recruitment rates, predation intensity on juvenile mussels, predator abundance, and finally the relative importance of recruitment and predation intensity on the abundance of mussels.

## Methods

### Study sites

**Figure 1:**
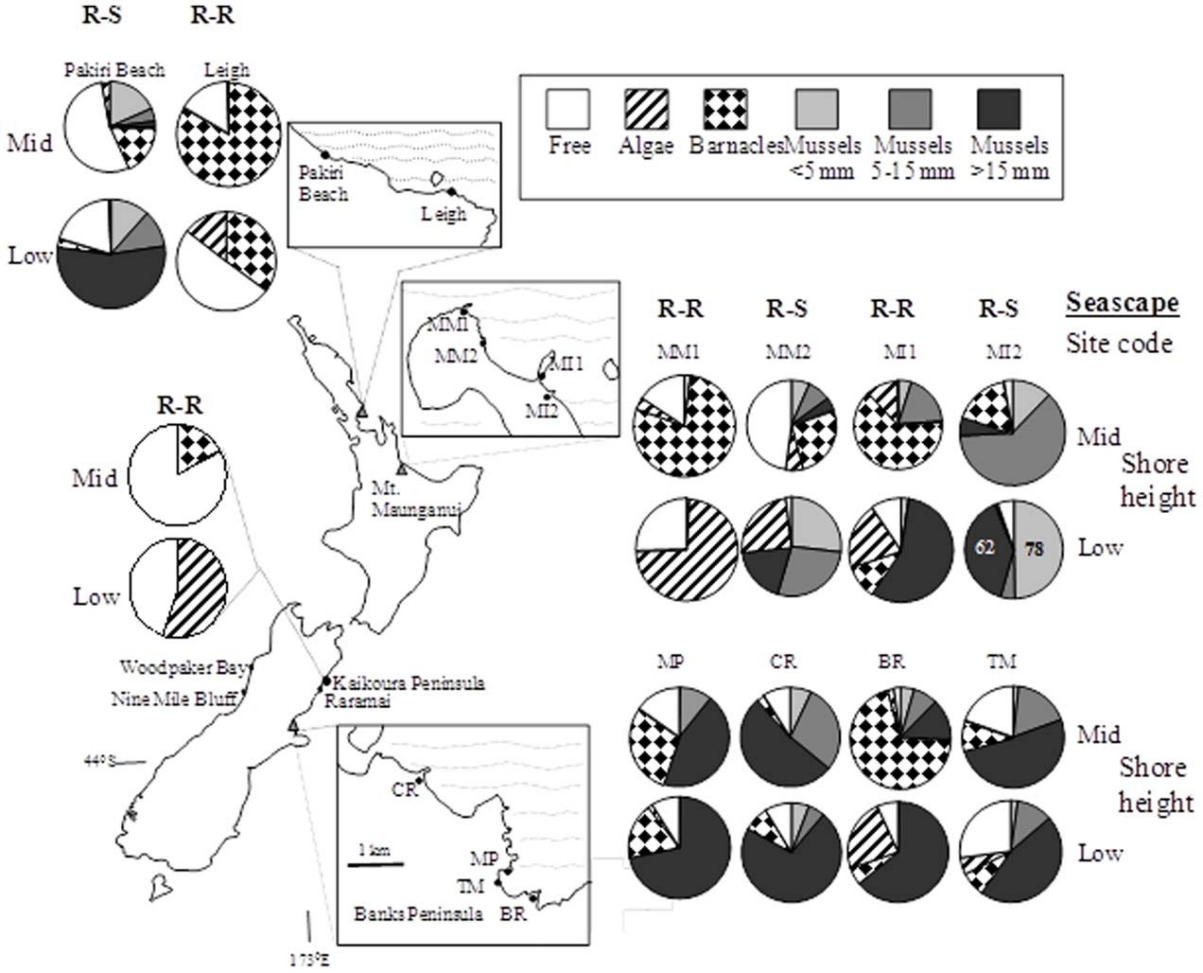
Study sites and percent rock cover of major space occupiers in the mid and low shore levels at R-R and R-S seascapes in New Zealand. Space occupiers include mussels of different size classes, macroalgae, barnacles and free space. CR is located about 1 km west of the nearest extensive rocky shore. TM is located 190 m from the western rocky side of a small sandy bay. MM2 is a small isolated bench located about 40 m from the rocky shore that surrounds the mount. MI2 is a rocky bench 200 m east of the Moturiki Island (see text). Pakiri is a R-S site which is at the beginning of a rocky shore at the end of the sandy Pakiri Beach. At the MI2 low shore, total cover exceeded 100% (because many mussel recruits covered adults) and actual percent cover for those groups is given in numbers on the pie chart. The Kaikoura Peninsula site was sampled in January 2004, Banks Peninsula sites in April 2004, Mt Maunganui sites in June 2004 and the Leigh sites in September 2004. A subset of this data appeared in Rilov and Schiel 2006a.

Eleven intertidal sites were used (Fig. 1). Two areas (each with four sites) enabled us to compare community structure (in the low and mid shore), predation intensity (low shore) and prey recruitment rates (low shore) between R-R or R-S seascapes within small regions. Three additional sites (one in the South Island and two in the North Island) were used to increase sample size for some aspects of this study. Site data and types of data taken in each site are given in Table S1 as well as tide levels.

The assumption was that all four sites located within a 2–3 km region (in the North and South Islands) are exposed to the same offshore larval pool [13], and any variability in local larval recruitment onshore is the product of differences in very nearshore or onshore processes. In the first area, the northwestern corner of Banks Peninsula on the South Island's east coast, there were two R-R sites (Moki Point, hereafter MP, and Black Rock, BR) and two R-S sites (Cave Rock, CR, and Taylor's Mistake, TM). In the second area, Mt. Maunganui in the Bay of Plenty on the North Island, there were two R-R sites (Mt. Maunganui 1, MM1, and Moturiki Island 1, MI1), and two R-S sites (Mt. Maunganui 2, MM2, and Moturiki Island 2, MI2, a rocky bench 200 m east of the Island). In both areas, sites were interspersed to avoid potential confounding effects due to spatial array. Of the three additional sites one was an R-R site on the South Island, Kaikoura Peninsula, where we quantified benthic cover and measured predation intensity at the low and mid shore levels and also measured mussel recruitment rates. The other two sites were in the North Island (Leigh, a R-R site, and Pakiri, a R-S site) where community structure (low, mid zones) and recruitment rates were quantified. All sites were moderately to highly wave-exposed.

### Benthic cover

Percent cover of the main space occupiers (mussels, barnacles, macroalgae) and of ‘free space’ was estimated during spring (North Island) and the end of the Austral summer (South Island) of 2004. Mussels were divided into 3 size classes: small (<5 mm, defined as recruits), medium (5–15 mm, defined as juveniles) and larger mussels (>15 mm, defined as adults). At sites where small mussels covered mature mussel beds, we recorded cover of both size classes, resulting in over 100% cover in some quadrats. Free space was defined as bare rock or rock covered by thin encrusting red algae [see, 29]. We used random-stratified sampling in the middle of the low and mid shore levels to estimate rock cover using twenty 20×30 cm quadrats along a 20–50 m transect at each shore level. We avoided deep crevices and rock pools in our sampling. Analysis was done separately on the data from each island because they were sampled in different seasons, which may have affected the cover of small mussel recruits (which were mostly rare in most sites at all dates). An assumption is that our single survey in each site gives a reasonable relative representation of mussel cover at the study sites and at least allows comparison among sites, seascapes and shore heights within a region (island).

### Predator presence and activity

#### Low tide surveys

Mobile invertebrate macro-predators (sea stars and whelks) were counted in the quadrats described above. We also conducted 10 min. number-per-unit-effort visual surveys in low shore cracks and crevices in the same area where quadrats were laid because these predators can be easily missed or underestimated using only the quadrats.

#### High tide surveys

Sampling the rocky intertidal zone during high tide on wave-swept shores could be done only when seas were relatively calm, recognizing this could also be when subtidal predators were most likely to forage in intertidal areas. At some sites (e.g., Banks Peninsula) we used prior data on the presence and activity of fish [28]. Fish presence and activity in different habitat types at the four North Island Mt. Maunganui sites were assessed during one afternoon high tide on August 2, 2004. Fish were counted by snorkeling using a 15 min. steady swim along and around rocks in the experimental areas. Three 5 min. observations (5 min. intervals between them) were used as replicates to record the number of predatory fish that visited an area (ca. 20 m^2^) and the number of bites that these fish took off the benthos around experimental plots. Two additional 5 min. snorkeling surveys were done at the four sites on August 30, 2004, at dusk (5:15–6:45 PM), four hours after the initiation of the predation experiment (see below), and in the morning (8:00–9:30 AM) of the next day. Numbers of sea stars and crabs (which are mostly nocturnal) were also recorded. The dusk survey included a record of predation activity on mussels on experimental tiles (see below). The predator surveys were intended to provide relative assessments of predator abundance and activity at study sites around the time of the experiments, and were not a thorough investigation of predator densities or temporal changes. Other, qualitative surveys (sometimes during harsher conditions) at R-R and R-S seascapes at different times supported the patterns we report below (Results section). However, we also recognize that within sites there can be great annual and inter-annual variation in densities mainly of recruits of these fishes [e.g., 30].

### Predation intensity experiments

The experimental units were comprised of 5–15 mm long mussels that were scraped from the rocks and settled on 5×5 cm carpet-covered plastic tiles that were secured to rocks with stainless steel screws [see], [ 27,28]. Twenty-five mussels were placed onto each tile, which was then wrapped with soft plastic mesh to secure the mussels until they were firmly attached by byssus threads (3–4 weeks). At the South Island sites, a mixture of *Mytilus galloprovincialis* and *Xenostrobus pulex* was used for prey because they were by far the most abundant small mussels in the area and are difficult to distinguish at small sizes. *M. galloprovincialis* was absent from North Island sites so only *X. pulex* was used. The experiments included transplanted mussels that were either exposed to the full suite of predators or protected by different types of cages to exclude different guilds of predators (see details in Table 1). It was logistically impossible to do the experiments in all sites at the same time, and the experimental design was somewhat different, depending on logistics and how much prior knowledge we had for each region (Table 1).

**Table 1:** Design of predation experiments.

Island	Area	Seascape	SL	Treatment	Species on tiles	Experiment duration
South	Banks Peninsula	2× R-R sites2× R-S sites	Low	No Cage	MG & XP	November 22, 2003–January 6, 2004
South	Kaikoura Peninsula	1 R-R site	Low+Mid	No CagePartial cageFull Cage	MG & XP	November 24, 2003–February 10, 2004
North	Mt Maunganui	2× R-R sites2× R-S sites	Low	No CageFull Cage	XP	August 30, 2004–September 4, 2004

R-R = reef-to-reef, R-S = reef-to-sand. MG = *Mytilus galloprovincialis*, XP = *Xenostrobus pulex*. SL = Shore level.

(At the South Island Banks Peninsula sites we used only tiles exposed to all predators (no-cage treatments) because we showed in a previous study that survival was always high for months inside full cages in this region at all seascape types (Rilov and Schiel 2006b). At Kaikoura we used no-cage, a partial-cage (3 cm gap at the bottom; excludes fish but not large invertebrate predators such as crabs, sea stars or whelks) and a full-cage (excludes all large predators) in both the low and mid shore levels. At the North Island Mt Maunganui sites we used the no-cage treatment and a full-cage control. Cage size was 10×10×10 cm, mesh gauge was 16×16 mm (for full details on cage design see Rilov & Schiel 2006a,b)).

### Prey recruitment rates

Mussel recruitment rates were measured at different sites using as substrate plastic-mesh ovoid pot scrubbers (SOS Tuffy Pads, The Clorox Company, CA, USA) that were fastened to rocks with stainless steel screws and washers (n = 5). Mussels attach to the filaments of the mesh balls that mimic the filamentous algal and mussel byssal thread surfaces that constitute common settlement sites in nature [e.g., 31] and it provides moisture and protection from most macro-predators. Many other studies that tested benthic-pelagic coupling models have used mussel recruitment to tuffies as a proxy for prey larval supply [e.g.], [ 20], [24,32]. At Banks Peninsula (South Island), settlement was monitored weekly or biweekly between January–December 2004, and at Kaikoura Peninsula (South Island), biweekly between December 2003 and May 2004. At the distant North Island sites, monitoring was done monthly between August–December 2004 (Mt Maunganui) and September–December 2004 (Leigh). Samples were stored in −20°C freezers prior to sorting and enumeration. In the lab, mussels were extracted from collectors using fast-flowing seawater, sieved and counted. Recruitment rate was expressed as mussels per collector per day.

### Predation-recruitment-mussel cover and predator abundance relationships

To test these relationships, we used all data from this study complemented by some data from earlier studies (Rilov and Schiel 2006a, 2006b), and by mussel recruitment data collected by B. Menge using the same methods over the same time period at three sites (Woodpecker Bay, Nine Mile Bluff on the west coast, and Raramai on the east coast of the South Island) for which we conducted predation but no recruitment measurements (B. Menge, *personal communication*). All data types and sources are summarized in Table S1. Average daily recruitment rates were calculated for the main recruitment season for which data were available.

To standardize predation intensity to natural mortality rates, we used an index of interaction strength. There are several different ways interaction strength has been calculated in the past [see review, 33]. We chose a modified version of an index used by Connolly and Roughgarden [13] for which we had the available data: 
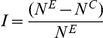
 where *I* is the interaction strength index, *N^E^* is the percent mortality (the inverse of survival) of small mussels on tiles exposed to all predators (experimental plot) and *N^C^* is mortality on tiles inside a cage (control plot). The index reflects the mussel mortality in plots exposed to all predators relative to protected plots and the values in this modified index would normally move between 0 (no interaction) and 1 (very strong interaction) but can also reach negative infinity if there is higher mortality for some reason inside than outside cages (this was never seen in our experiments). In this measure of predation intensity, the mean percent mortality for days 3 or 5 (depending on the experiment monitoring regime that was determined by logistics and sea conditions) for all predation experiments was used because most unprotected mussels were gone in R-R sites within the first few days of the experiment. We also looked for population-level effects by testing a variety of potential predictors for percent mussel cover (only mussels>15 mm, i.e., cover excluding recruits). These were 1) predation rate, 2) mussel recruitment, and 3) the combined effect of predator abundance and mussel recruitment. Data for whelks, fish and sea stars were pooled by functional group (i.e., whelks = all predatory whelk species, fish = the two labrid species (Banded wrasse, *Notolabrus fucicola* and Spotty, *Notolabrus celidotus*), sea stars = mostly *Stichaster australis* but also cushion stars if they were present). We have no fish data for the west coast sites (too rough and murky to sample) but labrids are known to be rare on the central parts of this coast where our study sites were located (Don Neale, New Zealand Department of Conservation, personal communication). We also have no data of fish abundances at KP or Raramai (on the east coast of the South Island) but labrids are known to be abundant on subtidal reefs in this area [34]. All the sites for which we had no fish data were not included in the correlations of mussels with fish abundance.

### Data Analysis

Abundance data from the community surveys were analyzed using 3-way mixed-model ANOVA (after arcsin square-root transformation) for each island (North, South) separately with seascape (R-S, R-R), and shore height (Mid, Low) as fixed factors, and site as a random factor nested within Seascape. Seascape, shore height and site were tested as main effects. Using data from the current study and a previous one ([28], plus unpublished data from R-S sites) we compared fish visitation rates (both labrid species combined) between islands (fixed factor), seascapes (fixed) and sites (random, nested in seascape) in a 3-way mixed-model ANOVA (after square-root transformation). Mussel percent survival in the predation experiment was tested using repeated-measures ANOVA after arcsin-square-root transformation. In the Banks Peninsula (South Island) and the Mt. Maunganui (North Island) experiments, seascape and site (nested in seascape) were tested with time as the within-subjects factor. In the Kaikoura Peninsula (South Island) experiment, shore-level and treatment were fixed factors and time was the within-subjects factor. To test the effect of seascape on recruitment we used data from all the sites for which we had information for the same period (September–December 2004). We used sites (6 in the North Island and 4 in the South Island) and sampling months (4 months) as replicates (n = 40) and tested the effect of seascape and island on recruitment (the calculated average recruitment per day for each site/month). Data were square-root transformed to satisfy the ANOVA assumptions. Stepwise multiple-regression analysis evaluated the relative contribution of mussel recruitment and interaction strength to percent cover of large mussels (>15 mm, because we are interested in how related these parameters are to the long-term patterns of the mussel populations). Residuals in probability plots indicated data were normally distributed. We also tested the correlation between mussel recruitment and interaction strength and also between predator abundance (whelks, sea stars, and fish) from our surveys and mussel recruitment rates and percent mussel cover.

### Ethics

All of our work was nondestructive except for the removal of several tens of juvenile mussels at each site for the predation experiment. At the time of this research, no permits or permissions were required for access and use of any of the field sites, or to work with the animals under study (mussels). Approval by the University of Canterbury Animal Ethics Committee was not required for this study. Where mussels were collected from the field, the amount taken was covered under a generic NZ Ministry of Fisheries collection permit, held by the School of Biological Sciences, University of Canterbury.

## Results

### Benthic cover

The most pronounced difference between R-R and R-S sites at both the North and South Island locations was the low coverage of mussels <15 mm (<2%) on the low shore at R-R sites and their much greater cover in the R-S sites (>50% in the North Island and >20% in the South Island, Fig. 1). However, there were considerable differences among sites within seascapes, which also varied by shore height. On the North Island, cover of all three mussel size classes varied with sites, seascapes and level on the shore (site(seascape)*shore-height interaction by size class: <5 mm, F_2,152_ = 15.6; 5–15 mm, F_2,152_ = 56.3; >15 mm, F_2,152_ = 26.4, p<0.0001 for all). On the South Island, a similar pattern occurred for the two smaller size classes (site(seascape)*shore-height interaction by size class: <5 mm, F_2,152_ = 6.3 p = 0.002; 5–15 mm, F_2,152_ = 4.1, p = 0.017) and seascape had a strong effect on the 5–15 mm size class (F_2,2_ = 320.2, p = 0.003). Generally, total mussel cover was higher in the R-S sites than at R-R sites in both the low and mid shore, except for the low shore at MP that had high cover of large mussels (Fig. 1). At MI2, mature *P. canaliculus* beds were covered mostly by *P. canaliculus* recruits, resulting in >100% cover in many quadrats. Mussel beds were absent from KP, where bare rock, macroalgae (low shore) or barnacles (mid shore) were abundant and individual mussels were found hidden in low zone crevices.

### Relative abundance and activity of predators

At low tide, whelk abundance and species composition varied greatly among sites and Islands with no outstanding differences between seascapes (for details see Table S2 in the online material). In most sites, there were more whelks in the mid than the low shore. In all locations, most individuals were found attached directly to rock during low tide and only a few were observed feeding. Small sea stars (*Stichaster australis*) were seen in crevices only in MM1 (North Island, R-R site). Crabs were rarely seen.

At high tide, whelks actively foraged, and sea stars were observed foraging out of cracks in the intertidal zone only in MM1 and at greater numbers after dark than in the daytime (5 and 2 per 5 min. survey, respectively). At the other North Island sites, sea stars were mainly seen in the subtidal zone, even during high tide. Large predatory crabs were rarely seen during daytime at high-tide, but at dusk, swimming crabs (*Ovalipes bipustulatus*) swarmed low and mid shore levels at the R-S site MI2 (59 were counted during a 5 min. survey) and actively fed on small mussels within and around the mussel bed, including the uncaged experimental mussels (see below). Crabs were not seen on MI1 rocks. Red rock crabs (*Plagusia chabrus*) occurred in the intertidal zone after sunset at the R-R site MM1 (10 individuals in a 5 min. survey). Predatory fish, *Notolabrus fucicola* (banded wrasse) and *Notolabrus celidotus* (spotty), were much more abundant at R-R than R-S sites (34–51 vs. 0–7 fish, respectively, per 15 min. swim), and R-R sites also had higher fish visitation (1.3–5.3 vs. 0–0.3 per 5 min.) and benthic foraging activity rates (0–1.3 vs. 0 bites per 5 min.) during daytime observations (for details see Table S3). Only “seascape” had a strong effect (F_1,18_ = 34.66, p<0.0001) when fish visitation was compared among islands, seascape and sites.

### Predation intensity experiments

**Figure 2:**
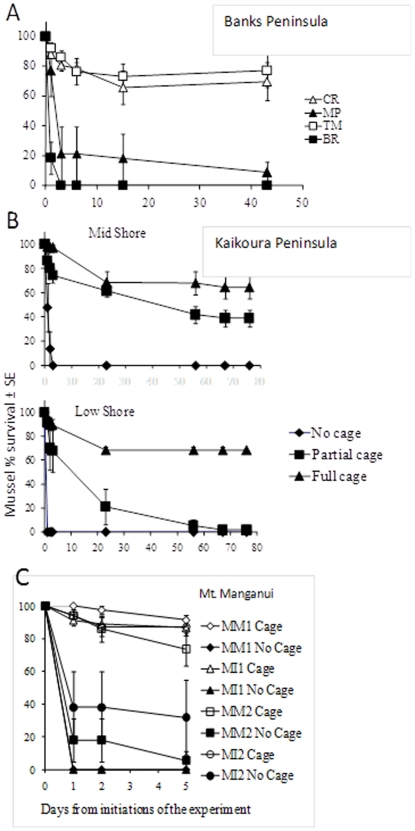
Effect of predation on mussel survival rates on R-S and R-R sites. (a) Banks Peninsula (sites: R-S = CR, TM, and R-R = MP, BR), only no-cage treatment and only low-shore level. (b) Kaikoura Peninsula, R-R site, low- and mid-shore levels, full cage control, partial cage and no cage treatments. (c) Mt. Maunganui, four sites (two R-S: MM2, MI2 and two R-R: MM1, MI1), both cage control and no-cage treatment; only low-shore level. Blank symbols = R-S sites, black symbols = R-R sites. Day 0 = day mesh was removed and mussels on tiles placed in the experimental treatments.

Predation on unprotected juvenile mussels was strong and rapid in all R-R sites throughout the study (Fig. 2). At the Banks Peninsula sites (South Island), the effect of seascape on survival was strong (F_1,15_ = 132.4, p<0.0001), with far greater survival (70–80%) at R-S sites (CR, TM) than at R-R sites (0–10%) after 45 days. There was also a strong time*seascape interaction (F_4,60_ = 4.7, p = 0.002) because survival remained high for the duration of the experiment in the R-S sites but was greatly reduced at the R-R sites (MP, BR, Fig. 2A). At the Mt. Maunganui sites in the North Island, there was a strong treatment effect (F_1,23_ = 118.8, p<0.001) and a weaker seascape*treatment effect (F_1,23_ = 7.1, p = 0.013) because predation on unprotected mussels (non-caged controls) was high compared to protected mussels (caged) at all sites, but was slower at the R-S (MM2, MI2) compared to the R-R (MM1, MI1) sites (Fig. 2C). A Time effect (F_2,46_ = 4.4, p = 0.017) and a weak time*treatment effect (F_2,46_ = 3.2, p<0.04) were also detected. At Kaikoura (R-R site), mortality on unprotected mussels was faster in the low than in the mid shore level (shore-level effect, F_1,24_ = 12.0, p = 0.002), varied among treatments (caging effect, F_2,24_ = 150.8, p<0.0001), and there was also a strong time*shore-level*treatment interaction (F_12,44_ = 5.2, p<0.0001), mainly because mortality in the partial cages was faster in the low than the mid shore level (Fig. 2B).

Most mussels disappeared from unprotected tiles within a day at R-R sites. Observations showed that most mussels were eaten during the first high tide after exposure to predation. Fish and crabs were often seen removing mussels within minutes of discovering them on experimental tiles during the first tide after the initiation of an experiment. In several instances, we witnessed that it took only several bites for labrid fishes to remove all the uncaged mussels from tiles in R-R sites in the North Island. Red rock crabs were seen feeding on mussels on the experimental tiles at dusk at R-R sites and swimming crabs were observed feeding on mussel bed and experimental mussels in one R-S site (MI2, North Island). No whelks or sea stars were seen eating the experimental mussels, even though whelks were abundant at most sites (Table S2 in online material).

### Prey recruitment rates

**Figure 3:**
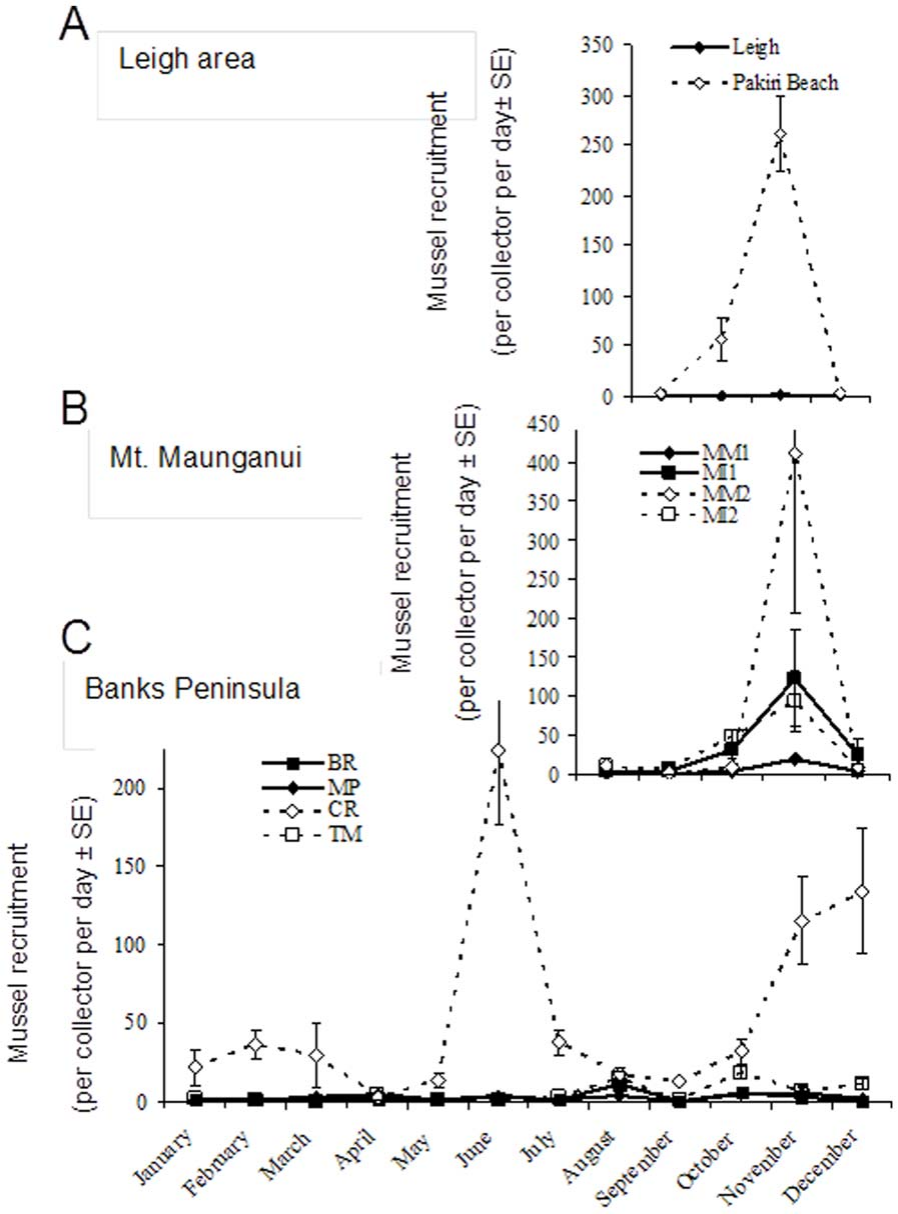
Mussel recruitment rates (mussels per collector per day) at R-R and R-S sites. From north to south (a) Leigh area sites, (b) Mt. Maunganui sites, (c) Banks Peninsula sites. Open symbols/dashed lines designate R-S sites and full lines / black symbols designate R-R sites. For Banks Peninsula sites we used data from a previous experiment [27] because in the current study we only used no-cage treatments in this region (not allowing the measurement of effect-size) but the results from the two experiments show very similar trends.

Mussel recruitment varied substantially with time in three regions (Fig. 3). The greatest recruitment was always in at least one (and sometimes both) R-S site in each region. At the R-R Kaikoura Peninsula site, only 7 mussels recruited to all collectors over a five month period. Analysis of data for the four months for which we had data for both the North and South Island sites shows that the average recruitment rates at R-S sites was 5.3 times greater than at R-R sites (61.5.8±23.4 SE and 11.5±6.2, per collector, respectively, two-way ANOVA, F_1,36_ = 7.36, p = 0.01) and the seascape effect was consistent between the two island (no island or seascape*island effects).

### Predation-recruitment-cover relationship

**Figure 4:**
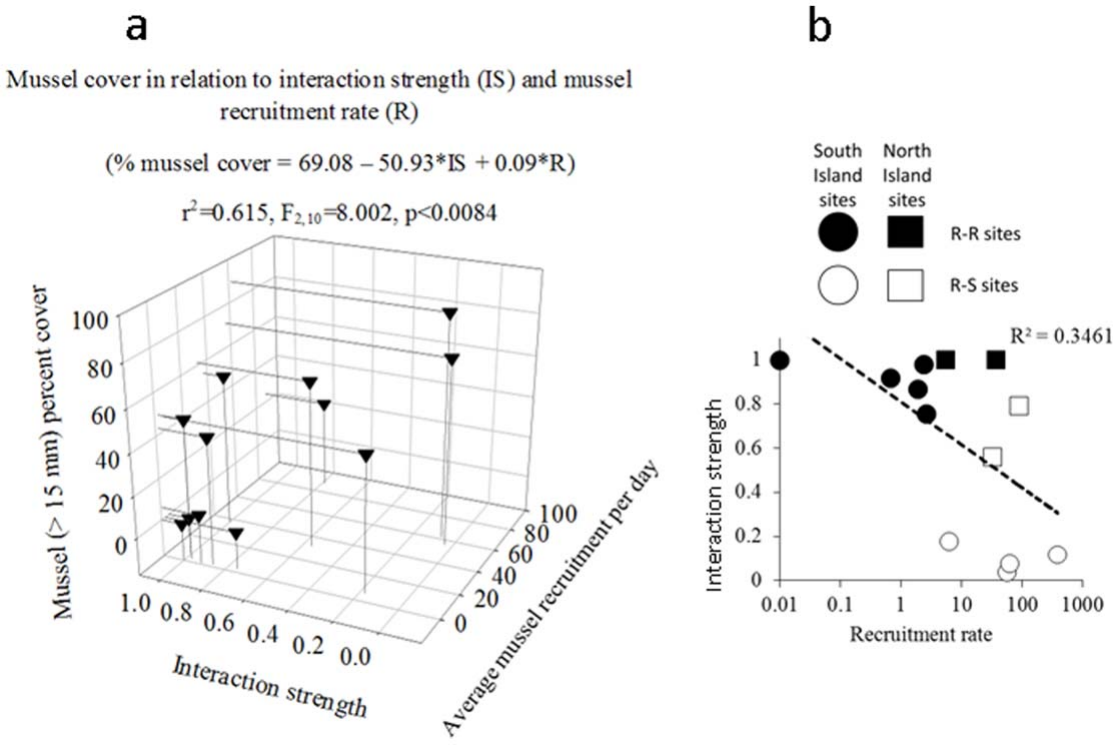
The relationship among recruitment rate, interaction strength and mussel percent cover on the rocks (a) and between recruitment rates (on a log scale) and interaction strength (b). For b, black symbols are R-R sites, blank symbols are R-S sites, circular symbols are South Island east coast sites, triangular symbols are South Island west coast sites, and square symbols are North Island east coast sites. Daily recruitment rates are calculated for August–December 2004 for the North Island and the east coast of the South Island sites, and October 2004–February 2005 for the South Island west coast sites.

**Figure 5:**
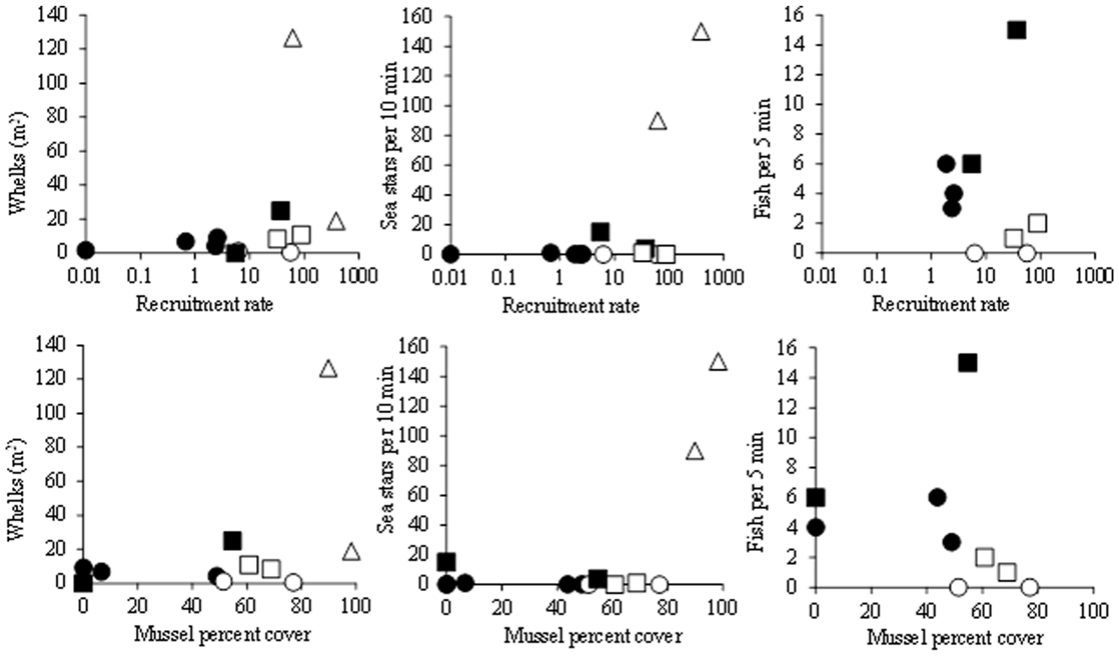
The relationships between mussel recruitment rates or mussel percent cover and predator abundance. Recruitment is per collector per day on a log scale and predator abundance is per m^2^ for whelks and per search time for sea stars and fish. Symbol shapes and fills as in Fig. 4.

Although both recruitment (positively, r = 0.59, p<0.05) and interaction strength (negatively, r = −0.74, p<0.05) strongly correlated with mussel (>15 mm) percent cover, stepwise multiple regression analysis indicated that interaction strength accounted for much more of the variability in mussel cover (beta = −0.59, p = 0.025) compared to recruitment rates (beta = 0.29, p = 0.21). When we run the analysis as an ANCOVA with Seascape as the categorical predictor variable none of the factors was significant as expected (because this relationship is driven by the seascape); however if we run the ANCOVA again with either forward or backward stepwise multiple regression, only interaction strength came up as a significant factor (p = 0.014). Fig. 4a shows that the lowest percent cover of mussels occurred where interaction strength was highest and recruitment was lowest. The relationship between recruitment rates and interaction strength (predation on juvenile mussels) was negative (r = −0.61, p<0.05, Fig 4b), but this relationship was mainly driven by seascape. Overall, R-R sites tended to have high predation intensity and low recruitment rates whereas R-S sites had weaker predation and higher recruitment (Fig. 4b). There were no apparent relationships (correlations) between intertidal predatory invertebrates or subtidal predatory fish and recruitment rates or percent mussel cover (Fig. 5). The lack of significant correlation with fish abundance is mostly due to one outlier site (Moki Point) with high abundance of fish and cover of mussels. This site seems more wave-exposed than the others and perhaps predation activity is reduced there allowing higher survivorship of mussels. Sea stars were found in their highest densities where mussel recruitment was also highest (west coast) but they were also rare or absent in other sites of relatively high mussel recruitment and cover.

## Discussion

The question of the relationship between food supply and predator abundance and predation intensity is fundamental in ecology. Answering this question becomes more difficult in marine systems where supply of young (both prey and predators) can be decoupled from birth (reproduction) rates due to the complex life cycle (pelagic larvae) of most benthic species. Earlier work suggested that prey recruitment and predation intensity might be decoupled by seascape characteristics [27] but there was not enough data to support it. The aim of the present study was to fill that gap.

Models that tested those relationships in upwelling systems predicted a strong positive correlation between prey supply rates and the consumption rates on them because most are influenced by the same oceanographic forcing [13]. Current evidence from the last decade suggests a more complex picture. There can indeed be a correlation between prey recruitment and predator abundance/predation-rate when both species are similarly affected by oceanographic forcing, for example in coral reef systems [21]. These relationships may not exist however if the species have very long or different pelagic larval duration, as was demonstrate in two large-scale studies on east Pacific coasts in both the north and south hemispheres [20], [23], [24]. But there can still be a relationship (just not driven by oceanography) if other factors determine predator abundance, such as a direct numerical functional response in non-dispersive predators [23], or the proximity of primary predator habitat determined by seascape that may also influence recruitment rates (this study; though the relationship turned out to be negative in this case).

In New Zealand, a comparative study on both sides of the South Island did suggest a strong coupling between coastal oceanography, mussel recruitment rates and predation pressure by sea stars [15]. In the same system, we show in this study that the recruitment-predation link is not general but rather highly context-dependent if the full suite of other predators that feed on smaller individuals is considered. We were also able to identify the mechanism responsible for the observed negative relationship between prey recruitment and predation intensity by showing that the levels of both processes depends on the characteristics of the reef seascape. The results for the current study indicate that predation intensity was a stronger predictor of the abundance of adults of a dominant space occupier (mussel beds) than recruitment rates.

Accounting for seascape in the study produced a negative correlation between mussel recruitment and predation intensity on small mussels. Rocky intertidal sites with low mussel recruitment and high predation intensity, and vice versa, exist, as do intermediate combinations (i.e., high-high or low-low, Fig. 4). The major predators on small mussels on the east coast of New Zealand are reef-dwelling labrid fish that are abundant on subtidal reefs at sites where intertidal mussels have very low recruitment levels and are extremely rare (e.g., KP, MM1). The consequence of this discrepancy is an intense and rapid predation on small mussels by these highly mobile predators, which eliminate the few mussels that manage to settle and grow to a juvenile stage at such R-R sites. This swift predation on small mussels can easily be overlooked in studies without fine-scale temporal resolution of post-recruitment predation. Notably, although we found some general patterns and processes at sites with similar seascapes (R-R or R-S), these were superseded by other site-specific features. For example, one R-S site had large numbers of swimming crabs that emerged from the adjacent sand at dusk and fed intensely on small mussels, indicating the potential for very localized, site-specific dynamics. In contrast to most studies on predation in intertidal communities, whelks played very little role in predation on small mussels, and in New Zealand, sea stars seem to be important only in the very low shore levels on some west coast benches (see, [29]).

Combining the data in this study and previous results on the effect of seascape on the presence and activity of subtidal predators [27], [28] allows generalizations to be made across a much greater spatial context. These studies show how fast-moving fish and crabs can forage intertidally on early life stages of intertidal habitat-dominating mussels, thus affecting their low-shore distribution and abundance. However, why seascape affects recruitment is still a matter of speculation. The greatest recruitment rates were always measured in at least one R-S site in each region and sometimes in both. Because R-R and R-S seascapes were paired within the same small region, they most likely shared a common nearshore pool of competent mussel larvae [32; Rilov unpublished data] and therefore differential availability of nearshore larvae between seascape cannot explain differential recruitment rates onshore. One suggestion is that the difference in recruitment rates between seascapes is a consequence of the combination of (1) the vertical positioning of competent mussels in the water column [in most cases, in both New Zealand and Oregon, the larvae are found in mid– rather than surface waters very close to shore, 32] and (2) the fact that intertidal mussel species can recruit and in some places form beds on subtidal reefs (e.g., *Perna canaliculus* is primarily a subtidal species). If this is true, then where subtidal reefs are present, a portion of the mussel larval pool may settle there and therefore be unavailable to the intertidal zone. Where subtidal reefs are absent, competent larvae have little choice but to settle intertidally. Some 23–26% and 31–32% of the recruitment to collectors on rocks at R-R and R-S sites, respectively, is made up of secondary settlers (>1 mm) compared to almost zero to collectors placed on moorings for similar durations (Rilov unpublished data). It is possible that the slightly higher percent of secondary settlers in R-S sites is also a result of scarcity of subtidal substrate in this seascape. The positive effect of limited substrate (e.g., due to sand cover) on intertidal settlement rates has previously been suggested for barnacles [35], [36], [see, 37]. Similarly, in estuaries, isolated appropriate habitat (oyster reefs in mudflats) was shown to increase juvenile fish recruitment compared to other (vegetated) areas [38]. Another plausible explanation for augmented recruitment on R-S sites could be that very local hydrodynamics are different between seascapes due to different bathymetry and R-S seascape which generates hydrodynamic conditions that facilitate settlement to the rocks. There are of course other possible explanations for the lower recruitment on R-R sites such as the “wall of mouths” idea [39], [40] where incoming mussel larvae are eaten by planktivores (e.g., fish) on the nearshore reefs in R-R sites before they can make it to shore, and thus intertidal recruitment is reduced at such sites.

**Figure 6:**
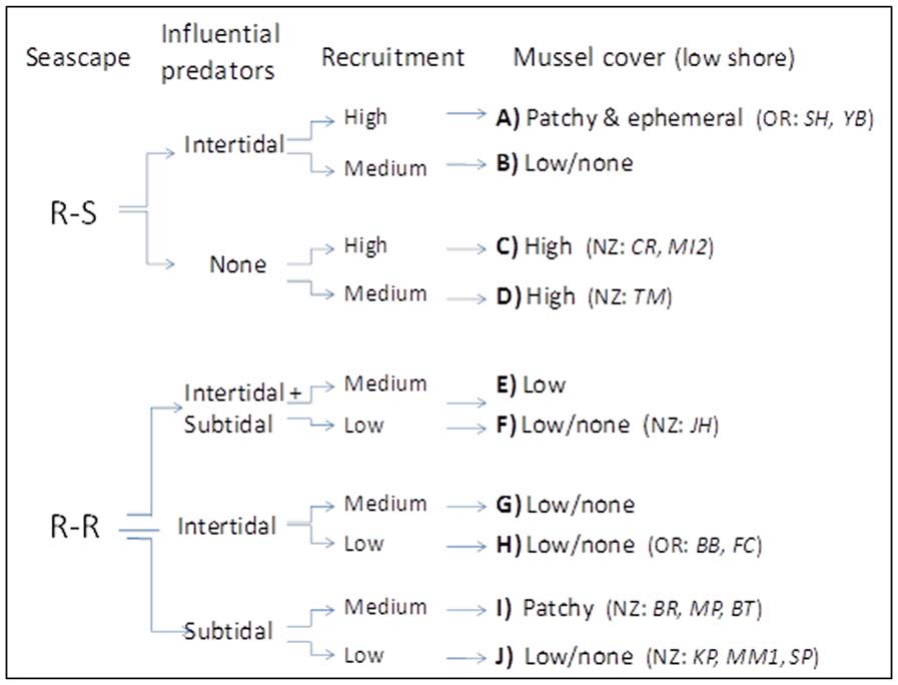
Conceptual model describing the influence of seascape, the presence of influential intertidal/subtidal predators (predators that have a strong influence on intertidal prey abundance) and recruitment on the distribution of mussel beds in the rocky shore in regions where mussel beds or patches are dominate features on the rocky shore (see text for explanation). The outcomes for the different scenarios are labeled alphabetically with bold capital letters. Examples of sites with the specific configuration are given in parentheses. Geographical regions: OR = Oregon (information from Menge publications and from Rilov unpublished data and personal observations), NZ = New Zealand. Site code names are from Fig. 1 and for Oregon sites as follows: SH = Strawberry Hill, YB = Yachats Beach, BB = Boiler Bay, FC = Fogarty Creek.

Here, a conceptual model is proposed (Fig. 6) that describes different predator-presence/absence scenarios (driven either by the regional assemblage of predators or by seascape) that could explain the observed distribution of mussels on rocky shores worldwide in the context of high (for R-S sites), medium (for R-S or R-R sites) or low (for R-R sites) mussel recruitment rates. Of course, not all situations may exist in all biogeographic regions because both shoreline topography and the regional predator guild can vary geographically, and mussels are not dominant intertidal organisms in all regions. At R-S seascapes located in biogeographical regions where intertidal predators (sea stars, whelks) are influential (i.e., can exert strong predation pressure) we expect patchy/ephemeral to very low mussel cover in the low shore depending on recruitment rates (scenarios A, B in Fig. 6; e.g., the Oregon coast at Cape Perpetua). Indeed, in Oregon R-S sites such as SH (see site name codes in Fig. 6 legend), or ones on New Zealand's central-northern west coast, temporary mussel beds can be formed in the low shore as the predators are first overwhelmed by the prey recruit numbers but over time eventually eat them [G. Rilov, personal observations, B. Menge personal communication, and see, 15]. Where intertidal predators are rare or not influential (e.g., whelks in some places such as New Zealand), mussel cover is expected to be high even when recruitment is low or medium because of low predation rates (scenarios C, D; e.g., R-S sites on the New Zealand east coast). In R-R seascapes, we expect mussel cover to be low or spatially patchy due to low recruitment rates. Where both intertidal (sea stars) and subtidal (fish, crabs) predators are present, mussel beds are restricted to mid-shore levels (scenarios E, F, probably the situation in JH on New Zealand southern west coast). Where only intertidal predators are influential we still expect low mussel cover because they usually feed on all mussel size classes (scenarios G, H, e.g., sites in Oregon at Cape Foulweather). Where intertidal predators are rare (or not influential) and subtidal ones present, low shore mussel beds are expected to be spatially patchy in medium recruitment sites (because most fish and crabs prey only on small mussels, mussels in microhabitats escape in size to form patches), and there is scarcity of mussel recruits between patches, or absence where recruitment is very low (scenarios I, J, e.g., most New Zealand east coast sites, [27]).

Although this proposed model describes processes on a local scale, these scenarios can also be scaled-up to coastlines, based on difference in geomorphologies among coasts. For example, in New Zealand, most rocky seascapes along the west coast of the North Island and the northern and central west coast of the South Island have subtidal benches with nearby subtidal sandy or pebble bottoms. Predatory reef fish such as labrids are rare in these habitats, probably due to the rarity of kelp beds there [34]. Most R-S benches are indeed covered by extensive mussel beds in the higher parts of the low shore and in midshore levels, with very high recruitment levels and occasional high densities of sea stars [29], [41]. In contrast, many parts of eastern New Zealand (both Islands) can be categorized as R-R seascapes with abundant predatory fish [34], [42] and very low mussel recruitment (Rilov unpublished data). At many of these sites, low shore mussel beds are patchy, scarce, or altogether absent. Dotted along the coast, often very close to R-R sites, R-S rocky benches occur, and mussel cover on them is frequently high, probably due to enhanced recruitment (see suggested mechanism above) and reduced predation.

The apparent non-linear “threshold” increase in sea star abundance beyond ca 100 mussel recruits per collector per day or ca 90% mussel cover (see Fig. 5) may be more a product of biogeography than of a threshold numerical response. The two sites with the highest sea star abundance were on the west coast where mussel recruitment and cover are indeed also high. Menge et al. [15] suggested that the different coastal oceanography on the two coasts (intermittent upwelling vs. downwelling) determines the pace of processes on the shore, and that “high abundances of prey may influence predator abundance by increasing survival of predator recruits, increasing predator growth rates…”. However, we found small isolated sites on the east coast with very high mussel recruitment rates but very low sea star abundances. Unless the numerical response, or more precisely the demographic response, is operating on a coastal scale the prey-predator abundance relationship alone does not hold, and other explanations for the significant difference in sea star abundances between coasts are required. The lack of a liner relationship between prey (mussel) recruitment/abundance and predator density fits well with the conclusion of Wieters et al. [23] who showed that such relationships are expected only for predators with non-pelagic larvae, and sea stars do have pelagic larvae.

**Conclusions.** Four major concepts have been stressed in this study: (1) predation can be more important than recruitment for the abundance of a dominant space occupier (mussels). (2) Local recruitment of prey and predation pressure on it (species interaction) can be negatively correlated because (3) seascape can greatly influence both larval recruitment rates of community dominants and predation on these community dominants, and therefore community structure in coastal systems – a good example of context-dependency. (4) The inclusion of interactions (e.g., predation) with the early life stages of benthic community dominants in experimental studies is crucial for the understanding of patterns and processes on the shore [see also, 43]. Furthermore, while mesoscale coastal processes (e.g., upwelling, eddies) may greatly contribute to regional variability in community structure [13], [15], [20], we demonstrate here that very local processes can result in just as much variability among sites with similar wave exposure within the same area. Including these concepts when designing future studies will increase the power of predictive models describing coastal community function and therefore help in achieving greater understanding of underlying processes.

Finally, the importance of landscape configuration to species interactions and how they affect communities have certainly become a focus of study in current ecological research. Recent examples come for instance from the African savanna where herbivore hotspots were related to landscape features that affect predation and nutrient input [44], and where movement and foraging activity of lions was related to landscape features and prey distribution [45], [46]. Our study contributes to this line of research by supplying evidence from a marine system on the importance of the local predator guild, the way it is affected by the local landscape, and the subsequent effects on community structure and processes. One can also view the intertidal-subtidal food-web linkage described in our study as a good example of an edge or refuge effect. Within this framework, forest edge effects are well known, and nest and seed predation rates in the forest have been linked to their proximity to an open area where mammalian nest predators come from [47], [48], [49]. Similarly, edge or refuge effects are known for marine environments. Lobsters living on New Zealand subtidal reefs, have been shown to fiercely forage on nearby sandy bottom prey [50], and in New England lobsters have been shown to move up to the intertidal at night to prey [51]. The next step is to find unifying or cross-ecosystem models that describe and explain these patterns and processes.

## Supporting Information

Table S1 **Site location, name, type of seascape, code name and type of information used in this study.** Mussel rock cover, recruitment and predation intensity was used for the multivariate analysis. The maximum tidal range on the east coast is ca. 2.4 meters. We define the low shore there as extending from 0–0.8 m (Lowest Astronomical Tide) and the mid shore from 0.8–1.6 m. The maximum tidal range on the west coast is ca. 3.7 meters. The low and mid shore zones on this coast extend from 0–1.2 m and 1.2–2.4 m, respectively.
(DOC)

Table S2 **Number of invertebrate macro-predators (whelks and sea stars) on rock surface (within 20×30 cm quadrats, numbers extrapolated to 1 m^2^) in the low and mid shore levels, and within low shore crevices (per 10 min. search time).** Surveys were conducted in the North Island on August 3, 2004, and the South Island on September 20, 2004.
(DOC)

Table S3 **Fish abundance (per 15 min. swim per site) and visitation (mean ± SD of three 5 min. observations of an area c 20 m^2^ near experimental plots) and feeding activity rates (mean ± SD of three 5 min. observations near experimental plots) at the four Mt. Maunganui sites.** Banded wrasse = *Notolabrus fucicola*, Spotty = .*Notolabrus celidotus*.
(DOC)

## References

[1] Agrawal AA, Ackerly DD, Adler F, Arnold AE, Caceres C (2007). Filling key gaps in population and community ecology.. Frontiers in Ecology and the Environment.

[2] Lewin R (1986). Supply-side ecology.. Science.

[3] Gaines S, Roughgarden J (1985). Larval Settlement Rate - a Leading Determinant of Structure in an Ecological Community of the Marine Intertidal Zone.. Proceedings of the National Academy of Sciences of the United States of America.

[4] Doherty P, Fowler T (1994). An empirical test of recruitment limitation in a coral-reef fish.. Science.

[5] Jenkins SR, Murua J, Burrows MT (2008). Temporal changes in the strength of density-dependent mortality and growth in intertidal barnacles.. Journal of Animal Ecology.

[6] Estes JA, Smith NS, Palmisano JF (1978). Sea otter predation and community organization in the western Aluetian Island, Alaska.. Ecology.

[7] Menge BA (2000). Top-down and bottom-up community regulation in marine rocky intertidal habitats.. Journal of Experimental Marine Biology and Ecology.

[8] Navarrete SA, Menge BA (1996). Keystone predation and interaction strength: Interactive effects of predators on their main prey.. Ecological Monographs.

[9] Silliman BR, Bertness MD (2002). Atrophic cascade regulates salt marsh primary production.. Proceedings of the National Academy of Sciences of the United States of America.

[10] Paine RT (1966). Food Web Complexity and species diversity.. The American Naturalist.

[11] Connell JH (1961). Effect of competition, predation by *Thais lapillus* and other factors on natural populations of the barnacle *Balanus balanoides*.. Ecological Monographs.

[12] Hixon MA, Carr MH (1997). Synergistic predation, density dependence, and population regulation in marine fish.. Science.

[13] Connolly SR, Roughgarden J (1999). Theory of marine communities: Competition, predation, and recruitment-dependent interaction strength.. Ecological Monographs.

[14] Fairweather PG (1988). Consequences of supply-side ecology - manipulating the recruitment of intertidal barnacles affects the intensity of predation upon them.. Biological Bulletin.

[15] Menge BA, Lubchenco J, Bracken MES, Chan F, Foley MM (2003). Coastal oceanography sets the pace of rocky intertidal community dynamics.. Proceedings of the National Academy of Sciences of the United States of America.

[16] Witman JD, Genovese SJ, Bruno JF, McLaughlin JW, Pavlin BI (2003). Massive prey recruitment and the control of rocky subtidal communities on large spatial scales.. Ecological Monographs.

[17] Menge BA (1995). Joint bottom-up and top-down regulation of rocky intertidal algal beds in South Africa.. Trends in Ecology & Evolution.

[18] Menge BA, Berlow EL, Blanchette CA, Navarrete SA, Yamada SB (1994). The keystone species concept: variation in interaction strength in a rocky intertidal habitat.. Ecological Monographs.

[19] Menge BA, Daley BA, Wheeler PA, Dahlhoff E, Sanford E (1997). Benthic-pelagic links and rocky intertidal communities: Bottom-up effects on top-down control?. Proceedings of the National Academy of Sciences of the United States of America.

[20] Navarrete SA, Wieters EA, Broitman BR, Castilla JC (2005). Scales of benthic-pelagic and the intensity of species interactions: From recruitment limitation to top-down control.. Proceedings of the National Academy of Sciences of the United States of America.

[21] White JW (2007). Spatially correlated recruitment of a marine predator and its prey shapes the large-scale pattern of density-dependent prey mortality.. Ecology Letters.

[22] Robles C, Sherwoodstephens R, Alvarado M (1995). Responses of a key intertidal predator to varying recruitment of Its prey.. Ecology.

[23] Wieters EA, Gaines SD, Navarrete SA, Blanchette CA, Menge BA (2008). Scales of dispersal and the biogeography of marine predator-prey interactions.. American Naturalist.

[24] Menge BA, Blanchette C, Raimondi P, Freidenburg T, Gaines S (2004). Species interaction strength: Testing model predictions along an upwelling gradient.. Ecological Monographs.

[25] Underwood AJ, Chapman MG, Connell SD (2000). Observations in ecology: you can't make progress on processes without understanding the patterns.. Journal of Experimental Marine Biology and Ecology.

[26] Menge BA, Branch GM, Bertness MD, Gaines SD, Hay M (2001). Rocky intertidal communities.. Marine Community Ecology.

[27] Rilov G, Schiel RD (2006). Seascape-dependent subtidal-intertidal trophic linkages.. Ecology.

[28] Rilov G, Schiel RD (2006). Trophic linkages across seascapes: subtidal predators limit effective mussel recruitment in rocky intertidal communities.. Marine Ecology Progress Series.

[29] Menge BA, Daley BA, Lubchenco J, Sanford E, Dahlhoff E (1999). Top-down and bottom-up regulation of New Zealand rocky intertidal communities.. Ecological Monographs.

[30] Jones GP (1984). Population ecology of the temperate reef fish *Pseudolabrus-Celidotus* Bloch and Schneider (Pisces, Labridae) .1. factors influencing recruitment.. Journal of Experimental Marine Biology and Ecology.

[31] Paine RT (1974). Intertidal community structure: experimental studies on the relationship between a dominant competitor and its principal predator.. Oceologia.

[32] Rilov G, Dudas S, Grantham B, Menge BA, Lubchenco J (2008). The surf zone: a semi-permeable barrier to onshore recruitment of invertebrate larvae?. Journal of Experimental Marine Biology and Ecology.

[33] Berlow EL, Navarrete SA, Briggs CJ, Power ME, Menge BA (1999). Quantifying variation in the strengths of species interactions.. Ecology.

[34] Schiel DR, Hickford MJH (2001). Biological structure of nearshore rocky subtidal habitats in southern New Zealand.. Science for conservation.

[35] Pineda J, Riebensahm D, Medeiros-Bergen D (2002). *Semibalanus balanoides* in winter and spring: larval concentration, settlement, and substrate occupancy.. Marine Biology.

[36] Pineda J, Caswell H (1997). Dependence of settlement rate on suitable substrate area.. Marine Biology.

[37] Pineda J (1994). Spatial and temporal patterns in barnacle settlement along a southern California rocky shore.. Marine Ecology Progress Series.

[38] Grabowski JH, Hughes AR, Kimbro DL, Dolan MA (2005). How habitat setting influences restored oyster reef communities.. Ecology.

[39] Gaines SD, Roughgarden J (1987). Fish in offshore kelp forests affect recruitment to intertidal barnacle populations.. Science.

[40] Hamner WM, Jones MS, Carleton JH, Hauri IR, Williams DM (1988). Zooplankton, planktivorous fish, and water currents on a windward reef face - Great Barrier-Reef, Australia.. Bulletin of Marine Science.

[41] Paine RT (1971). A short-term experimental investigation of resource partitioning in a New Zealand rocky intertidal habitat.. Ecology.

[42] Francis MP (1996). Geographic distribution of marine reef fishes in the New Zealand region.. New Zealand Journal of Marine and Freshwater Research.

[43] Osman RW, Whitlatch RB (2004). The control of the development of a marine benthic community by predation on recruits.. Journal of Experimental Marine Biology and Ecology.

[44] Anderson TM, Hopcraft JGC, Eby S, Ritchie M, Grace JB (2010). Landscape-scale analyses suggest both nutrient and antipredator advantages to Serengeti herbivore hotspots.. Ecology.

[45] Valeix M, Loveridge AJ, Davidson Z, Madzikanda H, Fritz H (2010). How key habitat features influence large terrestrial carnivore movements: waterholes and African lions in a semi-arid savanna of north-western Zimbabwe.. Landscape Ecology.

[46] Hopcraft JGC, Sinclair ARE, Packer C (2005). Planning for success: Serengeti lions seek prey accessibility rather than abundance.. Journal of Animal Ecology.

[47] Donovan TM, Jones PW, Annand EM, Thompson FR (1997). Variation in local-scale edge effects: Mechanisms and landscape context.. Ecology.

[48] Kollman J, Buschor M (2003). Edges effects on seed predation by rodents in deciduous forests of northern Switzerland.. Plant Ecology.

[49] Wassmann P (2011). Arctic marine ecosystems in an era of rapid climate change.. Progress in Oceanography.

[50] Langlois TJ, Anderson MJ, Babcock RC (2005). Reef-associated predators influence adjacent soft-sediment communities.. Ecology.

[51] Jones PL, Shulman MJ (2008). Subtidal-intertidal trophic links: American lobsters [Homarus americanus (Milne-Edwards)] forage in the intertidal zone on nocturnal high tides.. Journal of Experimental Marine Biology and Ecology.

