# Supplementary material for: Community Regulation: The Relative Importance of Recruitment and Predation Intensity of an Intertidal Community Dominant in a Seascape Context
Source: PLoS One. 2011 Aug 26;6(8):e23958. doi: 10.1371/journal.pone.0023958 (PMC3162600; doi:10.1371/journal.pone.0023958)
Supplement: Table S1 — Site location, name, type of seascape, code name and type of information used in this study. Mussel rock cover, recruitment and predation intensity was used for the multivariate analysis. The maximum tidal range on the east coast is ca. 2.4 meters. We define the low shore there as extending from 0–0.8 m (Lowest Astronomical Tide) and the mid shore from 0.8–1.6 m. The maximum tidal range on the west coast is ca. 3.7 meters. The low and mid shore zones on this coast extend from 0–1.2 m and 1.2–2.4 m, respectively. (DOC) [file pone.0023958.s001.doc]

| **Island** | **Coast** | **Region** | **Seascape** | **Lat (S)** | **Long (E)** | **Site name** | **code name** | **Rock cover** | **Mussel recruitment** | **Predation intensity** | **Invert. predator abundance** | **Subtidal Predator activity** |
| --- | --- | --- | --- | --- | --- | --- | --- | --- | --- | --- | --- | --- |
| South | East | Banks Peninsula | R-R | 43°35'1.36" | 172°47'1.06" | Black Rock | BR | **X** | **X** | **X** |  |  |
| South | East | Banks Peninsula | R-S | 43°34'54.19" | 172°46'34.99" | Taylors Mistake | TM | **X** | **X** | **X** | **X** | **X**** |
| South | East | Banks Peninsula | R-R | 43°34'49.22" | 172°46'41.02" | Moki Point | MP | **X** | **X** | **X** | **X** | **X**** |
| South | East | Banks Peninsula | R-S | 43°33'56.10" | 172°45'35.40" | Cave Rock | CR | **X** | **X** | **X** | **X** | **X**** |
| South | East | Kaikoura Peninsula | R-R | 42°26'51.16" | 173°34'59.92" | Raramai | RR | **X** | **X*** | **X** | **X** | **X**** |
| South | East | Kaikoura Peninsula | R-R | 42°25'28.92" | 173°43'3.09" | Kaikoura Peninsula | KP | **X** | **X** | **X** |  |  |
| South | West | Central West Coast | R-S | 42° 1'54.71" | 171°22'41.91" | Woodpecker Bay | WB | **X** | **X*** | **X** |  |  |
| South | West | Central West Coast | R-S | 42°20'57.44" | 171°15'17.77" | Nine Mile Bluff | 9MB | **X** | **X*** | **X** |  |  |
| North | East | Mt. Maunganui | R-R | 37°37'33.95" | 176°10'32.25" | Mt Maunganui 1 | MM1 | **X** | **X** | **X** | **X** | **X** |
| North | East | Mt. Maunganui | R-S | 37°37'45.69" | 176°10'36.25" | Mt Maunganui 2 | MM2 | **X** | **X** | **X** | **X** | **X** |
| North | East | Mt. Maunganui | R-R | 37°37'54.80" | 176°11'5.54" | Moturiki Island 1 | MI1 | **X** | **X** | **X** | **X** | **X** |
| North | East | Mt. Maunganui | R-S | 37°38'0.44" | 176°11'10.91" | Motukiri Island 2 | MI2 | **X** | **X** | **X** | **X** | **X** |
| North | East | Leigh | R-R | 36°16'8.27" | 174°47'55.94" | Leigh | LH | **X** | **X** |  |  |  |
| North | East | Leigh | R-S | 36°15'28.78" | 174°44'55.18" | Pakiri beach | PKB | **X** | **X** |  |  |  |

**X = Data from the current work, X* = Data supplied by Bruce Menge, X** data from Rilov and Schiel 2006a**
