# Supplementary material for: Community Regulation: The Relative Importance of Recruitment and Predation Intensity of an Intertidal Community Dominant in a Seascape Context
Source: PLoS One. 2011 Aug 26;6(8):e23958. doi: 10.1371/journal.pone.0023958 (PMC3162600; doi:10.1371/journal.pone.0023958)
Supplement: Table S2 — Number of invertebrate macro-predators (whelks and sea stars) on rock surface (within 20×30 cm quadrats, numbers extrapolated to 1 m2) in the low and mid shore levels, and within low shore crevices (per 10 min. search time). Surveys were conducted in the North Island on August 3, 2004, and the South Island on September 20, 2004. (DOC) [file pone.0023958.s002.doc]

| North Island | | | | | | | | | | | | | |
| --- | --- | --- | --- | --- | --- | --- | --- | --- | --- | --- | --- | --- | --- |
| Seascape | reef-to-reef | | | | | |  | reef-to-sand | | | | | |
| Site | MM1 | | | MI1 | | |  | MM2 | | | MI2 | | |
| Habitat | Low | Mid | Crevices | Low | Mid | Crevices |  | Low | Mid | Crevices | Low | Mid | Crevices |
| Units | per m^2^ | | per 10 min | per m^2^ | | per 10 min |  | per m^2^ | | per 10 min | per m^2^ | | per 10 min |
| Whelks |  |  |  |  |  |  |  |  |  |  |  |  |  |
| *Buccinulum spp.* | 0 | 0 | 16 | 1.7 | 0 | 3 |  | 0 | 2.5 | 1 | 0 | 0 | 0 |
| *Haustrum haustorium* | 0 | 0 | 3 | 0 | 0 | 0 |  | 0 | 0 | 0 | 0 | 0 | 0 |
| *Haustrum scobina* | 0 | 3.3 | 3 | 0.8 | 70.8 | 3 |  | 0 | 2 | 0 | 0 | 89.2 | 0 |
| *Dicathais orbita* | 0 | 0 | 2 | 22.5 | 2.5 | 67 |  | 10.8 | 0.8 | 31 | 8.3 | 13.3 | 95 |
| Sea stars |  |  |  |  |  |  |  |  |  |  |  |  |  |
| *Patiriella spp.* | 0 | 0 | 2 | 0 | 0 | 4 |  | 0 | 0 | 0 | 0 | 0 | 1 |
| *Stichaster australis* | 0 | 0 | 13 | 0 | 0 | 0 |  | 0 | 0 | 0 | 0 | 0 | 0 |
|  | | | | | | | | | | | | | |
| South Island | | | | | | | | | | | | | |
| Seascape | reef-to-reef (R-R) | | | | | |  | reef-to-sand (R-S) | | | | | |
| Site | MP | | | BR | | |  | CR | | | TM | | |
| Habitat | Low | Mid | Crevices | Low | Mid | Crevices |  | Low | Mid | Crevices | Low | Mid | Crevices |
| Units | per m^2^ | | per 10 min | per m^2^ | | per 10 min |  | per m^2^ | | per 10 min | per m^2^ | | per 10 min |
| Whelks |  |  |  |  |  |  |  |  |  |  |  |  |  |
| *Buccinulum spp.* | 0 | 0 | 1 | 1.7 | 1.7 | 2 |  | 0 | 0 | 0 | 0 | 0 | 2 |
| *Haustrum haustorium* | 0.8 | 0 | 5 | 2.5 | 1.7 | 12 |  | 0 | 0 | 3 | 0.8 | 0 | 0 |
| *H. scobina* | 0 | 6.7 | 11 | 0 | 0 | 9 |  | 0.5 | 3 | 1 | 0.4 | 7.5 | 15 |
| *Paratrophon spp.* | 8.3 | 0 | 3 | 0 | 0 | 6 |  | 0 | 0 | 1 | 0 | 0 | 3 |
