# Supplementary material for: Community Regulation: The Relative Importance of Recruitment and Predation Intensity of an Intertidal Community Dominant in a Seascape Context
Source: PLoS One. 2011 Aug 26;6(8):e23958. doi: 10.1371/journal.pone.0023958 (PMC3162600; doi:10.1371/journal.pone.0023958)
Supplement: Table S3 — Fish abundance (per 15 min. swim per site) and visitation (mean ± SD of three 5 min. observations of an area c 20 m2 near experimental plots) and feeding activity rates (mean ± SD of three 5 min. observations near experimental plots) at the four Mt. Maunganui sites. Banded wrasse = Notolabrus fucicola, Spotty = .Notolabrus celidotus. (DOC) [file pone.0023958.s003.doc]

|  | Seascape | reef-to-reef (R-R) | | | |  | reef-to-sand (R-S) | | | |
| --- | --- | --- | --- | --- | --- | --- | --- | --- | --- | --- |
|  | Site | MM1 | | MI1 | |  | MM2 | | MI2 | |
|  | Fish species | Banded wrasse | Spotty | Banded wrasse | Spotty |  | Banded  wrasse | Spotty | Banded wrasse | Spotty |
| Abundance | Per 15 min, Aug. 2 | 9 | 25 | 13 | 38 |  | 0 | 0 | 5 | 2 |
|  | Pre 5 min, Aug. 31 | 2 | 4 | 5 | 10 |  | 2 | 0 | 0 | 1 |
| Visitation | Per 5 min, Aug. 2 | 1.3±0.9 | 1.7±0.7 | 3.3±0.9 | 5.3±0.3 |  | 0 | 0 | 0.3±0.3 | 0.3±0.3 |
| Feeding activity | Per 5 min, Aug. 2 | 0 | 0.3±0.3 | 1.3±0.9 | 1.3±0.3 |  | 0 | 0 | 0 | 0 |
